# *Ferula asafoetida* oleo-gum resin alleviates dyspepsia symptoms through modulation of microbiome-gut-brain axis: A randomized, double-blind, placebo-controlled study

**DOI:** 10.1097/MD.0000000000044590

**Published:** 2025-10-03

**Authors:** Syam Das S, Veena RM, PA Aneesa, Sherin Joy Parappilly, Johannah Natinga Mulakal, Baby Chakrapani PS, Krishnakumar Illathu Madhavamenon

**Affiliations:** a R&D Centre, Akay Natural Ingredients, Cochin, Kerala, India; b Department of Pharmacology, BGS Global Institute of Medical Sciences, Kengeri, Bangalore, India; c Centre for Neuroscience, Cochin University of Science and Technology, Cochin, Kerala, India; d Department of Biochemistry, Sree Sankara College, Kalady, Kerala, India.

**Keywords:** asafoetida, dysbiosis, FenuMat, functional dyspepsia, gut-brain axis, gut-microbiome, postprandial distress

## Abstract

**Background:**

Functional dyspepsia (FD) is a prevalent gut–brain interaction disorder that adversely affects cognitive function. Current treatment options offer limited efficacy and are often associated with undesirable side effects. Hence, in this study, we evaluated the efficacy of a food-grade, self-emulsifying hydrogel formulation of *Ferula asafoetida* oleo-gum resin (ASF) in improving dyspepsia symptoms, cognitive function, sleep quality, and gut microbiota in individuals with FD symptoms.

**Methods:**

A randomized, double-blind, placebo-controlled trial was conducted with 62 participants diagnosed with FD symptoms. Subjects received 250 mg/d of ASF or placebo for 14 days. Outcome measures included the Leuven Postprandial Distress Scale, choice reaction time test, Bergen Insomnia Scale (BIS), Bristol Stool Form Scale, and gut microbiome profiling.

**Results:**

ASF treatment showed a significant time, treatment, and treatment × time effect for early satiety, bloating, and heart burn (*P* < .05). Further analysis of Leuven Postprandial Distress Scale data by Mann–Whitney *U* test provided the influence of ASF on days 1, 3, 7, and 14 which indicated a progressive improvement in the number of positive responders, especially for bloating, early satiety, and postprandial fullness (*P* < .05). ASF also significantly modulated the gut microbiota by decreasing the Firmicutes-to-Bacteroidetes ratio (71.9%; *P* < .001), enhancing alpha diversity (*P* < .05), enriching beneficial genera (e.g., *Bacteroides, Prevotella*), and reducing harmful taxa (e.g., *Escherichia, Clostridia*). Further, ASF demonstrated improvements in digestion and reduced constipation as indicated by the Bristol Stool Form Scale, with type 1 stools decreasing from 65% to 18% and type 2 from 35% to 7% by day 14. Neurocognitive assessments showed improved attention and focus (44% reduction in reaction time, *P* < .001), while BIS results indicated better sleep quality (ΔBIS = 10.89 ± 3.23, *P* < .001) on day 14.

**Conclusion:**

ASF demonstrated significant modulation of the microbiome–gut–brain axis, resulting in reduced dyspepsia symptom severity, enhanced cognitive performance, improved sleep quality, and better digestive outcomes. These findings support the potential of ASF as a safe and effective dietary supplement for gut and cognitive health in individuals with FD.

## 1. Introduction

The association of human health with the gut has long been acknowledged, as Hippocrates in 400 BC said “Death sits in the bowels.” Post-genomic research has also delineated the role of gut, especially the intestinal microbiota, on human health.^[1]^ Human gut microbiota has a complex ecosystem with approximately 2 million bacteria belonging to about 1000 microbial species (both beneficial and pathogenic).^[2]^ There exists a balance between the good and bad ones, and any disturbance in this balance, known as gut dysbiosis, may lead to diseases.^[3–6]^ While age, diet, lifestyle, and environmental factors play critical role in shaping the gut microbiome, usage of antibiotics, neuronal injuries, toxins like *Helicobacter pylori*, obesity, and stress lead to dysbiosis.^[7–9]^ It has been demonstrated that the enteric nervous system of the digestive tract can communicate with various organs in the body through a bidirectional network of signaling pathways mediated by microbiomes or its metabolites and is important for maintaining systemic homeostasis.^[7–10]^ Such a communication of the gastrointestinal (GI) tract with the central nervous system is known as gut-brain axis (GBA), also known as microbiome-gut-brain axis (MGBA). Gut dysbiosis can affect physiology, including the brain functions such as sleep, and cognition.^[11–14]^

Functional dyspepsia (FD) is one of the most commonly diagnosed disorders of gut-brain interaction worldwide and is reported to affect about 40% of global population.^[15,16]^ Dyspepsia symptoms are common adverse effects associated with glucagon-like peptide-1 (GLP-1) drugs (semaglutide, dulaglutide, exenatide, etc).^[17]^ The delay in gastric emptying and peaks of the GLP-1 effect with short-acting formulations have been hypothesized as causative agents for GI disturbances.^[18,19]^ The most commonly cited methodology for managing side effects of GLP-1 drugs is individualized and gradual dose-escalation of the medication, and if the symptoms are persistent or severe, needs to stop. Although the exact mechanism is not well understood, the pathophysiology of FD is complex.^[20]^ On a macroscopic scale, gastric physiological factors including gastroesophageal reflux, delayed or rapid gastric emptying, gastric dysrhythmias (motility disorders), or changes in mucosal and immune functions may attribute to FD.^[15,21,22]^ In a biochemical point of view, impaired barrier function, gastroduodenal inflammation, and/or *H pylori* infection may lead to dysbiosis and hence the FD.^[20,23,24]^

According to Rome IV criteria, FD composed of both postprandial distress syndrome (PDS) (bloating, early satiation, postprandial fullness) and epigastric pain syndrome, such as epigastric pain and heartburn.^[16,20]^ Current therapeutic strategies for FD are primarily focused on symptomatic treatment using proton pump inhibitors to suppress gastric acid secretion, motility agents, anxiety/depression drugs, as well as drugs for *H pylori* eradication.^[25]^ Though drugs can provide interim benefits, they are not recommended for supplementation due to possible side effects.^[26]^ There has been a growing trend in the use of complementary and alternative medicine, particularly herbal and spice-based therapies, among individuals with FD highlighting its perceived therapeutic benefits.^[16]^ However, the impact of such botanicals on gut microbiomes has rarely been exploited.

Recently, it was shown that consumption of Indian curry with mixed spices can modify/restore gut microbiome in 24 to 48 hours.^[27]^ Hingu (oleo-gum resin of *Ferula asafoetida*) is a generally recognized as safe-listed kitchen spice and medicinal herb widely used as a food flavor additive for centuries. *F asafoetida* is an herbaceous plant (*Umbelliferae* family) native to Asia and is commercially growing in Iran and Afghanistan. The oleo-gum resin, a milky exude or latex, is tapped from 4 to 5-year-old plant roots by making a wound, during March to May period.^[28]^ It has been widely used in traditional Indian, Chinese, and Arab system of medicine for digestive issues.

Various preclinical studies on asafoetida have suggested its plausible therapeutic potential in gut and brain function. Asafoetida has improved memory and cognitive performance in d-galactose/sodium nitrite-induced memory impaired mice.^[29]^ In another study, asafoetida showed efficacy in ameliorating peripheral neuropathy.^[30]^ It has also improved learning and memory in Wistar rats, potentially through cholinesterase inhibition and antioxidant mechanisms.^[31]^ With respect to GI pharmacology, asafoetida has exhibited smooth muscle relaxant activity in the GI tract.^[32]^ It has also modulated digestive enzyme activity in albino rats.^[33]^ Further, coadministration of asafoetida with curcumin has ameliorated inflammatory bowel disease in rats, with significant anti-inflammatory effect.^[34]^ Previous clinical evidences are also supporting the ameliorating role of asafoetida in FD.^[28,35]^

Considering the positive effects of asafoetida in gut and brain, the present study was aimed to investigate its influence on dyspepsia symptoms, and further to evaluate its effect on GBA modulation. The study followed a randomized, double-blind, placebo-controlled design with a short-term supplementation of a food-grade formulation of asafoetida oleo gum resin (14 days, 250 mg × 1/day). We hypothesized that asafoetida would be modulating gut microbiome to support a healthy gut brain axis. Since FD can affect both digestive and cognitive functions like sleep and attention, symptom questionnaires, neurocognitive tests, and gut microbiome analysis were employed in the study. To improve taste and ease of use, a self-emulsifying hydrogel formulation (ASF) developed with fenugreek (*Trigonella foenum-graecum*) mucilage was employed.^[36]^

## 2. Methods

### 2.1. Study design

**Figure 1.**
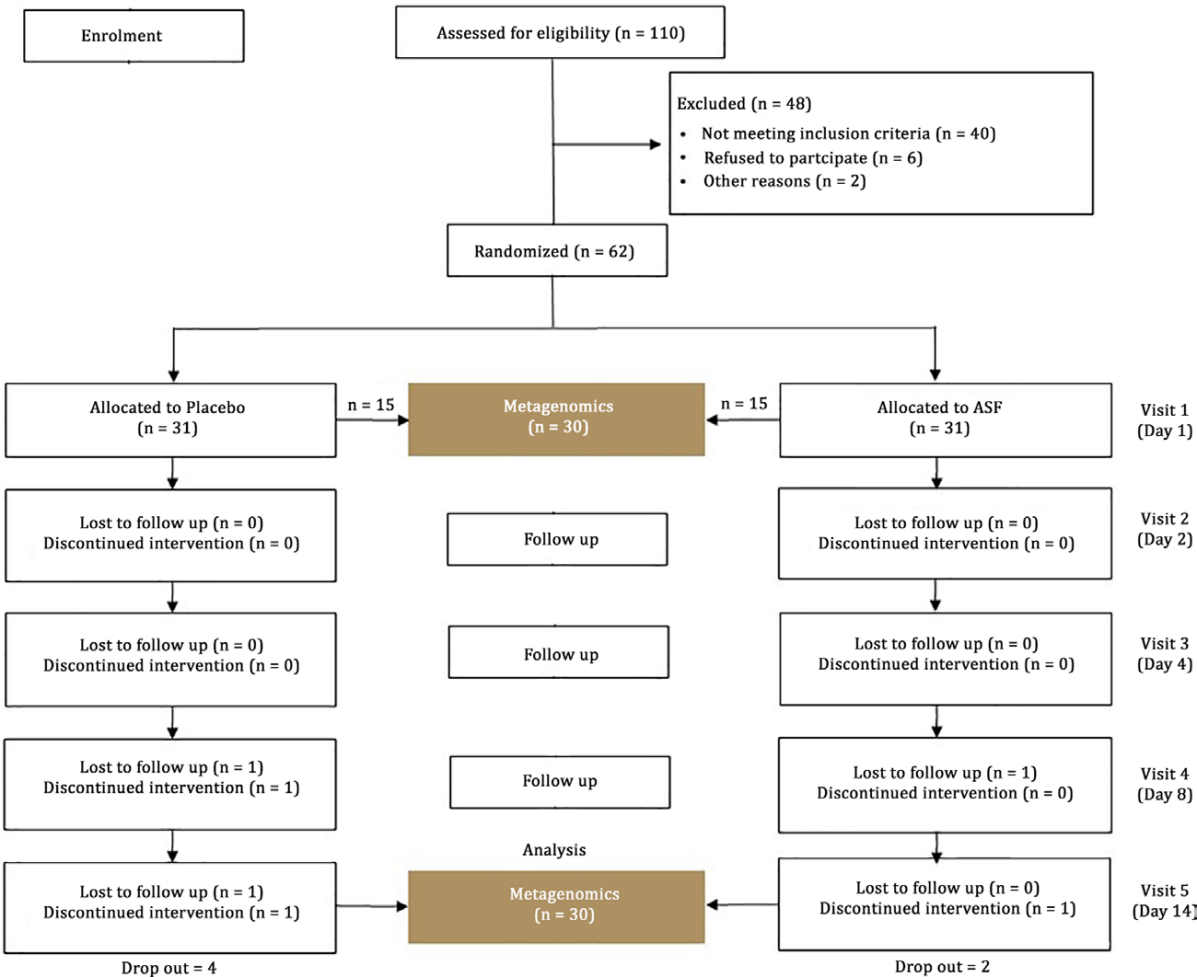
CONSORT diagram illustrating the study design. ASF = Asafin.

The study involved a randomized, double-blind, placebo-controlled design in accordance with the Declaration of Helsinki (Fig. 1). The institutional ethical committee reviewed and approved the protocol and was registered in the Clinical Trial Registry of India (CTRI/2022/07/044075, dated July 18,2022). A written informed consent for voluntary participation was also obtained prior to the commencement of the study.

### 2.2. Participants and sample size

Healthy volunteers (males and females aged 18–75 years) who have reported experiencing mild to moderate FD symptoms were selected from the data base of study center and nearby hospitals with the prerequisite of ROME IV criteria for FD. Sample size was calculated using G power statistical software (3.1.9.7 Version, Franz Faul, University of Kiel, Kiel, Germany).^[37]^ To achieve 80% statistical power with a 5% significance level and an anticipated 20% noncompliance or dropout rate, each study arm would require a total of 31 participants.

A total of 110 volunteers were screened, and 62 participants meeting the inclusion and exclusion criteria were initially selected for the study (Table 1). They were provided with comprehensive information about the trial, and an online screening questionnaire consisting of questions to assess general health, food pattern, medication usage, alcohol, drug consumption (including nicotine), and dietary supplements. Participants were instructed to maintain their usual diet, physical activity, and lifestyle behaviors throughout the 14-day intervention period. Those who were pregnant, using regular medications or dietary supplements, or had recent antibiotic exposure, were excluded from the study to minimize confounding factors.

**Table 1:** Inclusion and Exclusion criteria.

Inclusion Criteria	Exclusion Criteria
Age 18–75 years	Having a major gastrointestinal disease other than FD such as peptic ulcer, gastrointestinal bleeding, history of malignancy, etc
Male and female subjects	Participants who have taken antibiotics in the last 4 weeks
Fulfilling Rome IV diagnostic criteria for functional dyspepsia	Those who are allergic to herbal supplements
Female subjects who are nonpregnant, non-lactating, surgically sterilized, or using a medically acceptable form of birth control, as determined by the investigator	Subjects with abnormal haematological or biochemical parameters
Willingness to give informed consent and comply with the study procedures	

FD = functional dyspepsia.

### 2.3. Intervention

The proprietary formulation of ASF and placebo were obtained from M/s Akay Natural Ingredients, Kochi, India, along with a detailed certificate of analysis indicating the relative composition of the chemical constituents and its food grade status in accordance with United States Food and Drug Administration requirements for dietary supplements. Microcrystalline cellulose flavored to feel like asafoetida was used as placebo. Both ASF and placebo were supplemented as identical hard shell gelatin capsules in airtight high-density polythene containers of similar size, shape, and label. Each bottle contained 28 capsules, and the subjects were advised to take 1 capsule before breakfast, 250 mg × 1/day. They were also allowed to take additional capsules in case of any dyspepsia symptoms, in such a way that the intake should not be more than 2 capsules per day. At the end of the day, the remaining capsules in each bottle were counted to estimate the compliance. The identity of ASF was confirmed by high performance thin layer chromatography with a reference standard for Iranian asafoetida, and the voucher specimen was deposited at the Herbarium of M/s Akay Natural Ingredients Private Limited (AK-ASF-01/22). The volatile oil content was measured using a standardized and approved method by the American Spice Trade Association (1997). Ferulic acid standard was obtained from Sigma-Aldrich, Bangalore, India, and was estimated using high-performance liquid chromatography, employing Shimadzu model LC 20 AT instrument equipped with M20A photodiode array detector (Shimadzu Analytical India Private Limited., Mumbai, India) and reverse-phase C18 column (250 × 4.6 mm, 3 μm).

### 2.4. Randomization and blinding

Participants were randomized during visit 1 (day 1), using computer-generated randomization codes, into 2 groups to receive either placebo or ASF for 14 days. The master randomization list prepared by an independent statistician was handed over to the pharmacist for the purpose of double blinding.

### 2.5. Outcome measures

The primary objective of the study was to evaluate the cumulative effect of ASF on day 3, 7, and 14 as per Leuven Postprandial Distress Scale (LPDS), along with measurement of relative changes in microbiome via metagenomics analysis, and the assessment of changes in stool type using the Bristol Stool Form Scale (BSFS). Since FD symptoms are known to affect brain functions through GBA, the secondary objective was to evaluate attention and focus using choice reaction time (CRT) test and sleep. Bergen Insomnia Scale (BIS) was employed to monitor sleep quality.

### 2.6. Study protocol

In a typical protocol, the participants were directed to attend the study location on 5 separate occasions: visit 1 (day 1/baseline), visit 2 (day 2), visit 3 (day 4), visit 4 (day 8), and visit 5 (day 14/end of study). During visit 1, participants underwent medical examinations including demography and anthropometric measurements and were randomized into either the placebo or ASF group. Baseline scores for LPDS, CRT, and BIS were also recorded on Day 1. LPDS data were also collected on day 2 (first day single dose data), day 4 (cumulative effect of first 3 days’ data), day 8 (cumulative effect of first 7 days), and day 14 (cumulative effect of 13 days). BSFS data were acquired on days 1, 8, and 14, and the microbiome analysis and CRT were carried out at the baseline (day 1) and at the end of the study (day 14). Blood (10 mL) was withdrawn on day 1 and day 14 for the analysis of plasma safety parameters. To minimize circadian variation and ensure consistency, all assessments were conducted during the morning hours (7:00 am–11:30 am). A schematic representation of the various activities during the visits is given in Figure 1.

### 2.7. Leuven Postprandial Distress Scale

LPDS is a sensitive and reliable patient-reported outcome instrument to assess symptoms associated with FD and PDS. It asks for 8 most common symptoms of dyspepsia (early satiety, postprandial fullness, upper abdominal bloating, epigastric pain, epigastric burning, nausea, belching, and heartburn), each scored on a 5-point scale (0 = symptoms absent, and 4 = very severe symptoms), with higher scores indicating greater severity.^[38]^

### 2.8. Fecal sample collection and metagenomics analysis

An at-home stool collection protocol was used for the meta-genomics study.^[39]^ A sterile collection tube, tissue papers, and gloves were provided to each participant along with instructions on proper stool sample collection and handling protocol to ensure hygiene, avoid cross-contamination, and maintain sample integrity. Each collection tube had a spatula attached to its cap to collect ~1 g of stool. The tubes were tightly closed, sealed in the large zip bag, and transported to the lab on dry ice. Each tube was labeled with a unique ID, vortexed for 5 seconds, and archived at −80°C.

QIAamp Fast DNA Stool Mini Kit (Qiagen, Hilden, Germany) was used to isolate DNA from stool samples. The purity of the extracted DNA was measured using Varioskan Lux (Thermo Scientific, Waltham). The V3-V4 regions of the 16S ribosomal RNA gene were amplified and subjected to 250 × 2 paired-end sequencing on Illumina HiSeq 2500 platform. Sequence reads were quality-filtered using the QIIME2 tool (version 2020.8) (Caporaso Lab, San Diego). The quality-filtered reads were processed into amplicon sequence variants and assigned taxonomy based on the SILVA database with a 95% similarity threshold. Amplicon sequence variants (ASVs) were rarefied to an even depth, and data processing was performed using the phyloseq package in R (Stanford University, Stanford) .

### 2.9. Bristol Stool Form Scale

BSFS is a validated descriptive and visual scale narrating 7 types of stools with their images and definitions.^[40]^ The participants were given 7 definitions and 7 images and asked to match each definition to its suitable images in 2 to 3 minutes. Type 1 and 2 typifies constipation, type 3 and 4 ideal stools, type 5 for a precursor to diarrhea, and types 6 and 7 for diarrhea.

### 2.10. Choice reaction time task

CRT was conducted following the method outlined by Deary et al as an online computerized test.^[41]^ Participants responded by pressing the correct key corresponding to the cross that appeared in 1 of the 4 boxes on the computer screen. The inter-stimulus interval varied randomly between 1 and 3 seconds, for a total of 50 stimuli. The mean reaction time for correct responses were recorded. All subjects were thoroughly acquainted with the procedure, and a practice trial was provided to every subject before taking the test. A reduction in mean reaction time for correct responses (milliseconds) indicates increased speed of attention.

### 2.11. Bergen Insomnia Scale

BIS is a standardized questionnaire consisting of 6 questions related to sleep and tiredness over a period of 1 week or 1 month.^[42]^ The 6 items include difficulty in sleep initiation and maintenance, early morning awakening, non-restorative sleep, day-time impairment, and the dissatisfaction associated with sleep. These items are rated on an 8-item Likert scale and total composite scores range from 0 to 42.

### 2.12. Statistical analysis

Statistical analysis was performed using SPSS software (version 28.0, IBM Corp., Armonk). Data were first assessed for normality using the Shapiro–Wilk test. For repeated measures analysis, the assumption of sphericity was evaluated using Mauchly test, where sphericity was violated, and Greenhouse–Geisser corrections were applied.

A 2 × 5 repeated measures analysis of variance (ANOVA) was used for parametric measures to assess the effects of time and intervention. Multiple comparisons were adjusted using the Bonferroni correction. For nonparametric data, analysis was performed using propensity score matching, followed by the Friedman rank-sum test and pairwise Wilcoxon signed-rank test with Bonferroni correction. Changes from baseline over time within each group were assessed using the paired sample *t*-test, and between-group comparisons were made using the independent *t*-test for parametric data, and the Mann–Whitney *U* test for nonparametric data. Results are reported as mean ± standard deviation, and statistical significance was set at *P* < .05.

## 3. Results

### 3.1. Study design

In total, 56 participants completed the study and there were 4 dropouts from the placebo group and 2 from the ASF group, due to either the difficulty adhering to the protocol, unexpected travel, and/or unrelated health issue (fever). The details are depicted in the CONSORT diagram (Fig. 1). Baseline demographic characteristics of the participants are presented in Table 2, and showed no statistically significant differences between the placebo and ASF.

**Table 2:** Baseline demographic characteristics of participants.

Characteristics	Placebo			ASF		
	Baseline	End of study	*P* value	Baseline	End of study	*P* value
Age (yr)	47.00 ± 12.00		–	49.00 ± 11.00		–
Height (cm)	170.37 ± 3.87		–	170.41 ± 3.52		–
Weight (kg)	77.66 ± 6.40	77.70 ± 6.46	.78	72.24 ± 6.29	72.48 ± 6.30	.14
BMI (kg/m^2^)	26.75 ± 1.96	26.76 ± 1.98	.78	24.91 ± 2.51	25 ± 2.52	.14
Systolic BP (mm Hg)	122.74 ± 3.86	122.18 ± 3.87	.50	124 ± 5.78	124.03 ± 6.01	.98
Diastolic BP(mm Hg)	86.23 ± 7.12	84.51 ± 7.62	.32	85.51 ± 6.08	83.48 ± 5.48	.13

Values are presented as mean ± SD.

ASF = Asafin, BP = blood pressure, BMI = body mass index.

*P* < .05 was considered statistically significant.

### 3.2. Compliance

The compliance rate as evident from the count pill strategy and study diary was 100% in both groups, though there were 3 instances (from 2 volunteers) of consumption after breakfast. Eight volunteers from ASF and 14 volunteers from placebo consumed 2 pills per day multiple times, especially when they felt full after meal, bloating, and heartburn. While placebo did not find any benefit of multiple intakes per day, ASF provided relief within 1 to 2 hours after intake, especially with a little walking. About 11 volunteers from placebo sought to synthetic acid suppressor drugs (omeprazole or ranitidine) on multiple occasions during the study period, while ASF did not.

### 3.3. ASF alleviated PDS severity: LPDS analysis

#### 3.3.1. By repeated measures ANOVA

The influence of ASF on LPDS was analyzed by 2 × 5 mixed repeated measures ANOVA to assess changes in LPDS parameters over treatment and time (Table 3). The reliability of the LPDS was assessed using intraclass correlation coefficient (ICC). An ICC value > 0.9 was observed, which indicated acceptable reliability.

**Table 3:** Changes over 14-day intervention on LPDS sub-scores analyzed by 2 × 5 mixed RM ANOVA.

Parameters	Effects	*F* value	*P* value
Early satiety	Treatment	13.587	.001
	Time	14.352	.001
	Treatment × time	3.462	.024
Postprandial fullness	Treatment	9.874	.004
	Time	10.835	.001
	Treatment × time	1.717	.181
Bloating	Treatment	34.735	.001
	Time	13.325	.001
	Treatment × time	5.258	.004
Epigastric pain	Treatment	1.364	.253
	Time	5.033	.005
	Treatment × time	1.137	.364
Epigastric burning	Treatment	3.721	.065
	Time	2.815	.049
	Treatment × time	1.740	.176
Nausea	Treatment	0.515	.480
	Time	0.784	.548
	Treatment × time	1.046	.405
Belching	Treatment	5.271	.030
	Time	1.332	.288
	Treatment × time	2.946	.042
Heartburn	Treatment	8.797	.006
	Time	3.681	.019
	Treatment × time	1.814	.161

Changes in LPDS sub-scores upon treatment with ASF and placebo statistically analyzed using 2 × 5 mixed model repeated measures ANOVA. Results are provided as *F* value and *P* value at various effects.

LPDS = Leuven Postprandial Distress Scale, RM ANOVA = repeated measures analysis of variance.

Early satiety significantly decreased upon ASF supplementation compared to the placebo, across all time points, as indicated by a significant main effect of treatment, *F*(2, 56) = 13.587, *P* = .001, partial *η*^2^ = 0.343. There was also a significant main effect of time, *F*(2, 56) = 14.352, *P* < .001, partial *η*^2^ = 0.714, indicating that early satiety decreased over the course of the intervention. In addition, the treatment × time interaction was significant, *F*(2, 56) = 3.462, *P* = .024, partial *η*^2^ = 0.376, showing that the decrease in early satiety over time was greater for ASF than the placebo. Another symptom which showed overall significant effect (treatment, time, treatment × time) was bloating. The observed treatment and time effect for ASF were [*F*(2, 56) = 34.735, *P* < .001, partial *η*^2^ = 0.572] and [*F*(2, 56) = 13.325, *P* < .001, partial *η*^2^ = 0.699] respectively, with a treatment × time interaction of *F*(2, 56) = 5.258, *P* = .004, partial *η*^2^ = 0.478.

Postprandial fullness demonstrated a significant main effect of treatment [*F*(2, 56) = 9.874, *P* = .004, partial *η*^2^ = 0.275], time [*F*(2, 56) = 10.835, *P* < .001, partial *η*^2^ = 0.653], and treatment × time [*F* (2, 56) = 1.717, *P* = .181, partial *η*^2^ = 0.230]. Heartburn was another symptom with a significant treatment effect [*F*(2, 56) = 8.797, *P* = .006, partial *η*^2^ = 0.253] and time effect [*F*(2, 56) = 3.681, *P* = .019, partial *η*^2^ = 0.390]. Treatment × time effect was *F*(2, 56) = 1.814, *P* = .161, partial *η*^2^ = 0.240. Epigastric pain, epigastric burning, and belching showed mixed results. While time effect size was significant for epigastric pain [*F*(2, 56) = 5.033, *P* = .005, partial *η*^2^ = 0.467] and epigastric burning [*F*(2, 56) = 2.815, *P* = .049, partial *η*^2^ = 0.329], belching showed significance for both treatment [*F*(2, 56) = 5.271, *P* = .03, partial *η*^2^ = 0.169] and treatment × time [*F*(2, 56) = 2.946, *P* = .042, partial *η*^2^ = 0.339].

#### 3.3.2. By Mann–Whitney *U* test

The impact of ASF on LPDS scores (as revealed by Mann–Whitney test), on day 2 (single dose effect), day 4 (3 days’ effect), day 8 (7 days effect), and day 14 (end of study) compared to both baseline (on day 1) and placebo are provided in Table S1, Supplemental Digital Content, https://links.lww.com/MD/Q31. There were no significant differences between the groups at the baseline (day 1) (*P* > .05). Upon supplementation, the number of positive responders in various symptoms increased progressively from baseline to day 14 in ASF group (Figure S1a and b, Supplemental Digital Content, https://links.lww.com/MD/Q30).

##### 3.3.2.1. Three days’ effect

After the first dose on day 1, more than 60% of the participants shared the experience of a positive feel on ASF compared to placebo. On day 4, LPDS data corresponding to the cumulative effect of the first 3 days of treatment were gathered. The percentage of positive responders in ASF, as defined by >50% relief in symptoms were, 72% for bloating, 52% for fullness, and 64% for heartburn (Figure S1a and b, Supplemental Digital Content, https://links.lww.com/MD/Q30). Upon intergroup comparison, belching was found to be reduced significantly (*P* = .007). indicating an overall improvement in digestion (Table S1, Supplemental Digital Content, https://links.lww.com/MD/Q31).

##### 3.3.2.2. Seven days’ effect

On day 8, the relative percentage of the population in the ASF group who showed significant relief in various symptoms were postprandial fullness (63%), bloating (76%), epigastric pain and burning (58%), and heart burn (72%), which was statistically significant with respect to baseline and placebo (*P* < .05) (Figure S1a and b, Supplemental Digital Content, https://links.lww.com/MD/Q30). The observed effect of the above-mentioned symptoms as revealed by the intergroup comparison was also statistically significant for ASF group (Table S1, Supplemental Digital Content, https://links.lww.com/MD/Q31).

##### 3.3.2.3. Fourteen days’ effect

By end of the study (day 14), more than 70% of ASF participants reported significant reduction in dyspepsia symptoms compared to their baseline status. The most significant improvements reported by percentage of population with respect to baseline scores in ASF group were: early satiety (74%; *P* < .001), postprandial fullness (74%; *P* < .001), bloating (81%; *P* < .001), epigastric pain (75%; *P* < .001), belching (74%; *P* = .004), and heartburn (79%; *P* < .001) (Figure S1a and b, Supplemental Digital Content, https://links.lww.com/MD/Q30). Intergroup comparison revealed reductions of about 81%, 53%, 60%, 33%, and 59%, respectively, for ASF group compared to placebo for symptoms like early satiety (*P* < .001), postprandial fullness (*P* = .001), bloating (*P* < .001), belching (*P* = .015), and heart burn (*P* < .001), respectively (Table S1, Supplemental Digital Content, https://links.lww.com/MD/Q31).

### 3.4. Metagenomics analysis

**Figure 2.**
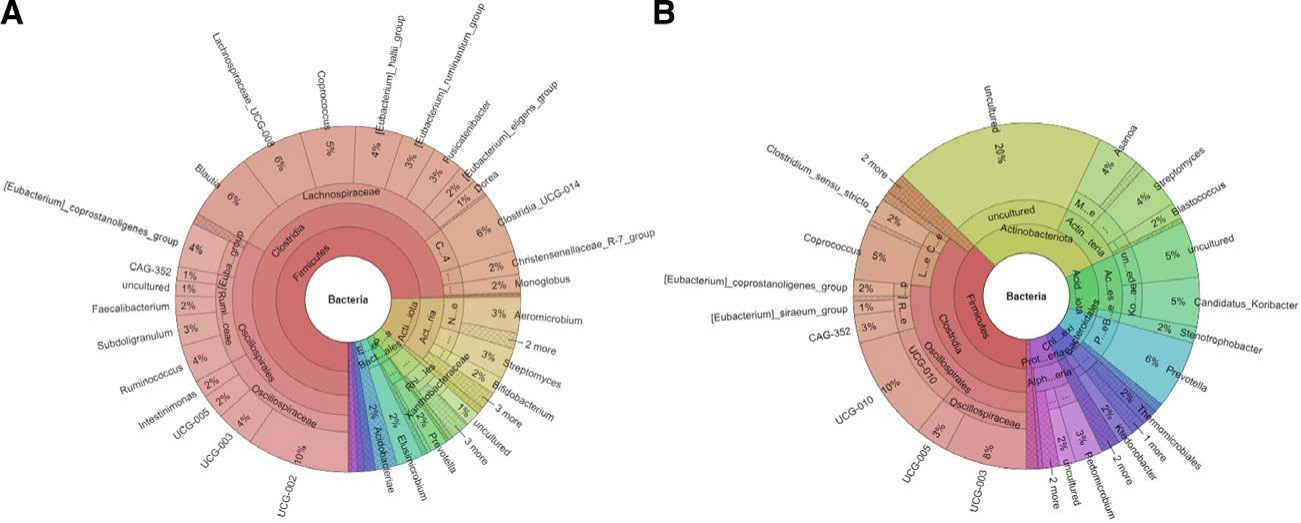
Krona plot of ASF group showing the taxonomic distribution (a) before treatment and (b) after treatment. The plot displays the hierarchical relationships between different microbial taxa, with the size of each sector representing the relative abundance of that taxon. Dominance in *Firmicutes* and reduction in *Privotellacea* are characteristic feature of FD. ASF = Asafin, FD = functional dyspepsia.

**Figure 3.**
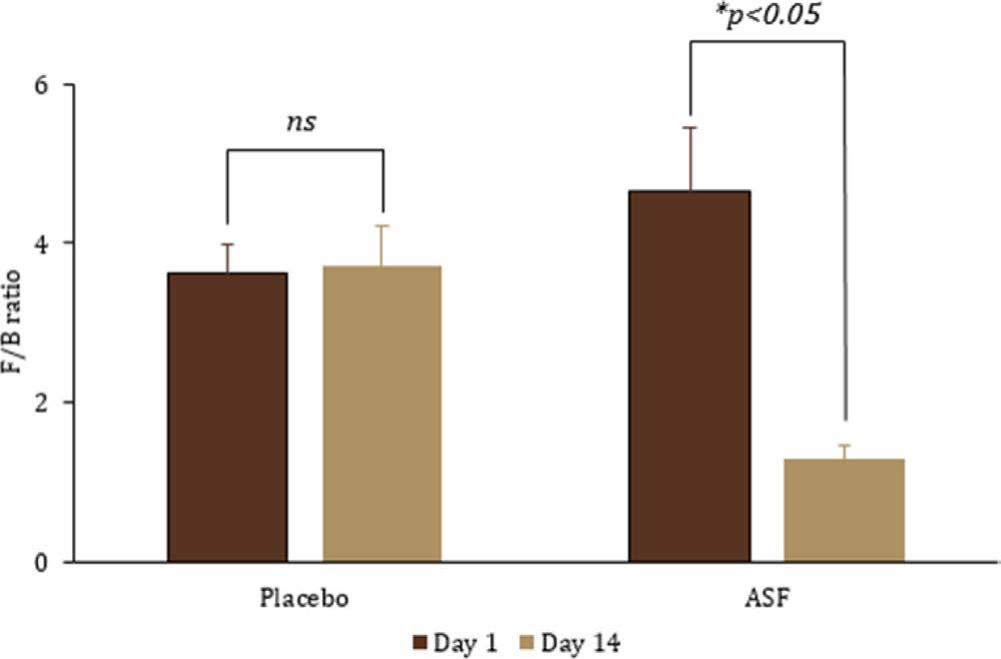
*Firmicutes*-to-*Bacteroidetes* ratio (F/B) upon ASF treatment. The values are expressed as mean ± SD. *P* < .05 indicates statistical significance. ASF = Asafin, SD = standard deviation.

Metagenomic analysis demonstrated remarkable differences in the relative abundance of various taxa across the 75% individuals supplemented with ASF for 14 days (Fig. 2). At phylum level, a significant reduction in *Firmicutes* was observed upon ASF treatment (treatment effect size: 0.973 [*F*(2,56) = 510.49; *P* < .001]; time effect size: 0.976 [*F*(2,56) = 569.63; *P* < .001]; time vs treatment effect size: 0.974 [*F*(2,56) = 526.82; *P* < .001]). The ASF treatment also showed a relative increase in *Actinobacteria*, followed by *Bacteroidota, Acidobacteria, Chlorofexi*, and *Proteobacteria*. Specifically, *Bacteroidetes* exhibited a treatment effect size of 0.639 [*F*(2,56) = 24.77, *P* < .001] and a time effect size of 0.514 [*F*(2,56) = 14.83, *P* = .002]. Among the *Firmicutes, Clostridia* was the predominant class in the placebo group, whereas its abundance was comparatively lower in the ASF-treated group. Notably, an increased prevalence of *Prevotella* was observed following ASF treatment. The *Firmicutes*-to-*Bacteroidetes* ratio (F/B) significantly reduced upon ASF treatment (4.64 ± 0.8 to 1.3 ± 0.16 [71.9%; treatment effect size: 0.650, *F* = 26.02, *P* < .001; time effect size: 0.872, *F* = 95.04, *P* < .001]) in comparison with placebo (Fig. 3).

**Figure 4.**
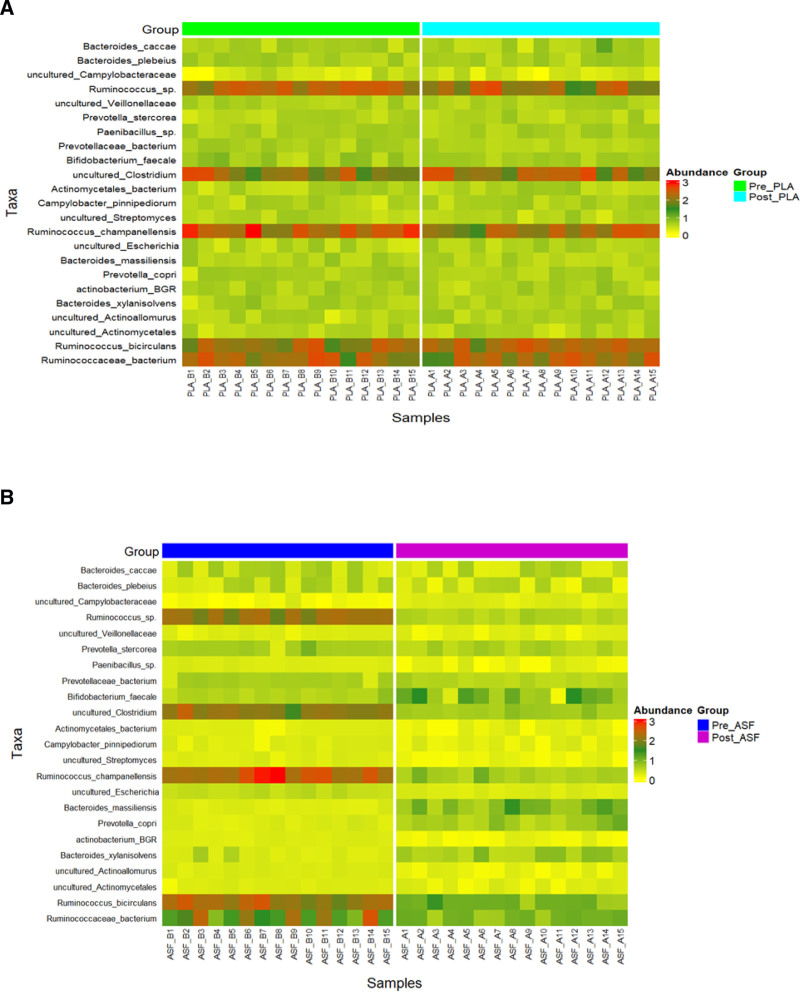
Complex heatmap showing relative abundance of major bacterial species within the samples of (a) Pre-PLA versus Post-PLA and (b) Pre-ASF versus Post-ASF. ASF = Asafin.

Comparative analyses were conducted across timepoints to evaluate microbiome composition before and after treatment in the same participants. Specifically, we compared microbiome profiles within the placebo (Pre-PLA vs Post-PLA) and ASF (Pre-ASF vs Post-ASF) to assess changes over time. The analysis revealed no significant changes in the microbial composition of the placebo group before and after the intervention, indicating a stable microbiome profile in the absence of treatment (Fig. 4a). The relative abundance of taxa, including *B faecale, P copri, P stercorea, P bacterium*, uncultured *Escherichia, R champanellensis, Ruminococcus* sp., and *R bicirculans* remained consistent across both time points in the placebo, with no statistically significant differences, suggesting that the administration of the placebo did not result in any substantial shifts in gut microbial composition between the Pre- and Post-placebo.

The evaluation of the differences between the 2 groups at baseline (Pre-PLA vs Pre-ASF) and at the treatment endpoint (Post-PLA vs Post-ASF) following the 14-day intervention was also carried out to determine treatment-specific alterations in the microbiome profile. Notably, a significant shift in microbial composition was observed in the post-ASF condition, suggesting that the treatment induced substantial alterations in the gut microbiota (Fig. 4b). ASF intervention led to a marked increase in the abundance of *B faecale, B massiliensis*, and *P copri*. Additionally, the abundance of *P stercorea* and *P bacterium* increased posttreatment; however, these changes did not achieve statistical significance. In addition to the enrichment of the above taxa, our results demonstrated a significant reduction in uncultured *Clostridia*, uncultured *Escherichia*, and several taxa within the genus *Ruminococcus*, specifically *R bicirculans* and *R champanellensis*.

### 3.5. Bristol Stool Form Scale

The reliability of BSFS was tested using the ICC, and a value above 0.9 showed that it was reliable. Analysis of stool types over the time, as measured by the BSFS, indicated a positive trend with more significant changes in ASF. The BSFS baseline values showed 65% type 1 and 35% type 2 in ASF group which changed to 26% type 1, 12% type 2, 58% type 3, and 4% type 4 by day 8. The changes were more prominent by day 14; 18% type 1, 7% type 2, 40% type 3, 35% type 4 (Table 4). So, a significant change in the stool type, with reduction in types 1 and 2 and increase in types 3 and 4 was observed. Though placebo also showed a positive trend, the changes were not significant compared to either the baseline or ASF group.

**Table 4:** Changes over 14-day intervention on Bristol scale ratings on days 1, 8, and 14.

Bristol scale ratings	ASF			Placebo		
	Baseline	Day 8	Day 14	Baseline	Day 8	Day 14
Type 1 (constipation)	65%	26%	18%	72%	58%	50%
Type 2 (constipation)	35%	12%	7%	22%	15%	32%
Type 3 (healthy)	–	58%	40%	–	12%	5%
Type 4 (healthy)	–	4%	35%	–	–	4%
Type 5 (prone to diarrhea)	–	–	–	6%	10%	9%
Type 6 (diarrhea)	–	–	–	–	6%	–
Type 7 (diarrhea)	–	–	–	–	–	–

Changes in the Bristol Stool Form Scales (BSFS) before and after the treatment with placebo and ASF for 14 days. The values expressed as percentage change.

ASF = Asafin.

### 3.6. Influence of ASF on speed of attention: CRT task

ASF supplementation revealed a significant treatment × time interaction for CRT [effect size: 0.680, 95% CI: 388.25–410.78, *F*(2,56) = 55.28, *P* < .001]. Post hoc analysis further revealed 45% reduction in reaction time compared to placebo on day 14 [treatment effect size: 0.738, 95% CI: 535.43–578.68, *F*(2,56) = 73.08, *P* < .001] (Table 5). Also, a significant reduction in reaction time on day 14 (44%), compared to the baseline [time effect size: 0.664, 95% CI: 535.94–585.94, *F*(2,56) = 51.39, *P* < .001] was observed for ASF. But, no improvement was observed in the placebo (*P* = .717).

**Table 5:** Changes in choice reaction time task on supplementation with placebo and ASF.

Groups	Choice reaction time (ms)		% change in reaction time	Treatment vs time		
	Day 1	Day 14		*P* value	*F* value	
Placebo	709.00 ± 114.65	722.37 ± 121.42	+1.88	<.001		55.28
ASF	713.59 ± 111.55	399.51 ± 28.48	−44.01			

Values are presented as mean ± SD. “+” indicates increase and “−” indicates decrease.

ASF = Asafin.

*P* < .05 was considered statistically significant.

### 3.7. Effect of ASF on sleep quality: BIS analysis

**Figure 5.**
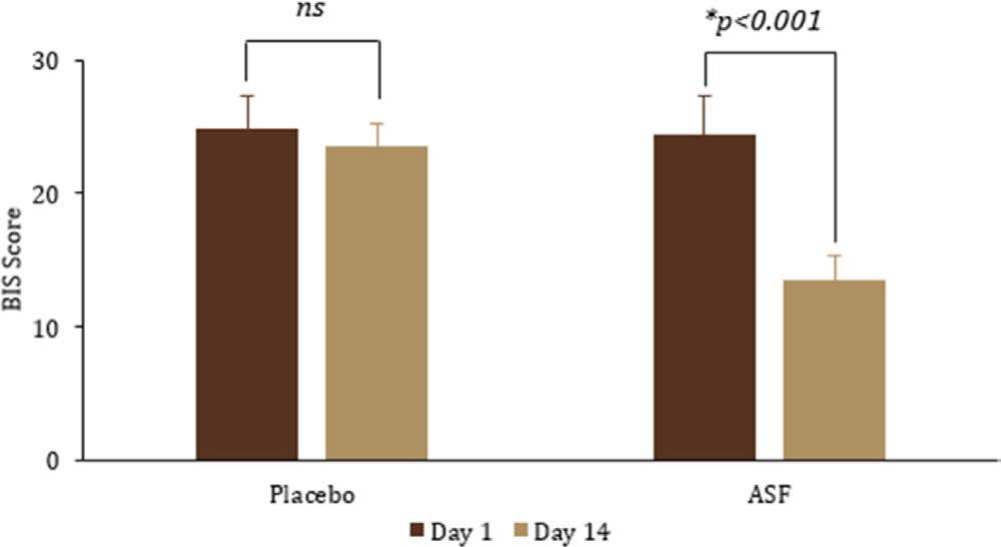
Effect of ASF on Bergen Insomnia Scale (BIS score). The values are expressed as mean ± SD. *P* < .05 indicates statistical significance. ASF = Asafin, SD = standard deviation.

Intragroup comparison of the mean difference in ASF showed a significant reduction in BIS scores by day 14 [ΔBIS = 10.89 ± 3.23; *P* < .001], while placebo exhibited no significant effect. Between group comparison also revealed a significant reduction in BIS score for ASF [ΔBIS = 10 ± 2.61; *P* < .001] compared to placebo (Fig. 5). Further, treatment × time interaction also exhibited a significant effect [effect size: 0.801, 95% CI: 12.793–14.170, *F*(2,56) = 104.59, *P* < .001].

### 3.8. Safety and Tolerance

Analysis of clinical safety parameters, especially liver, kidney, and hematological parameters did not show any safety issues (Table S2, Supplemental Digital Content, https://links.lww.com/MD/Q31). However, 7 volunteers reported the taste/smell of asafoetida in mouth, especially in the initial days. Though this was an inconvenience, it was not considered as significant when compared with the relief they experienced.

## 4. Discussion

Herein we report the very first human intervention study of a botanical to ameliorate FD and improve the quality of life through the modulation of GBA in a short duration (14 days). The study employed a food-grade formulation of asafoetida oleo-gum resin using fenugreek soluble dietary fiber as a soft hydrogel scaffold (ASF), at a dose of 250 mg/day for 14 days, to alleviate dyspepsia discomforts by modulating gut microbiome. In the study, healthy subjects having dyspepsia-like symptoms were selected and the influence of ASF was rated using LPDS questionnaire on day 1 and the cumulative effects of supplementation for 3 days, 7 days, and 14 days respectively. Out of the 29 participants in ASF group, 19 participants with baseline LPDS score >3 reported early satiety and postprandial fullness as their main concern. Similarly, 21 subjects reported bloating as the main issue. The relative number of subjects reported with other symptoms were in the order, epigastric pain (n = 12), epigastric burning (n = 11), belching (n = 15), and heart burn (n = 15).

The results of the study helped to better understand if ASF can provide a fast action on dyspepsia symptoms. Since dyspepsia has been identified as disorders of gut-brain interaction with significant impact on cognitive functions such as attention, reaction time, and sleep, we also attempted to monitor these functions. The rationale of the study was mainly a previous clinical study of Asafin on FD, and its positive effects on cholinergic and anti-histaminergic pathways, ability to relax GI smooth muscles, as well as adrenergic, purinergic, and gamma amino butyric acid effects.^[35,43]^

LPDS, one of the most used and well-validated self-reported rating scale, was employed to monitor the important components (i.e., early satiety, postprandial fullness, bloating, epigastric pain/burning, and heartburn) as a measure of the overall dyspepsia symptoms.^[38]^ A treatment × time interaction revealed a significant effect for ASF on the severity of various dyspepsia symptoms compared to placebo. Early satiety, bloating, and heart burn were the symptoms shown to be the most significantly affected by ASF with an overall time, treatment, and treatment × time effects. While epigastric pain, epigastric burning, and postprandial fullness showed significant effect over the time, ASF treatment did not exhibit a treatment or treatment × time interaction effect. However, ASF treatment showed no significant effect for nausea, compared to placebo.

Mann–Whitney *U* test of the LPDS data on days 2, 4, 8, and 14 showed that the severity of dyspepsia symptoms progressively decreased from baseline over the time period, for ASF compared to both baseline and placebo. About 70% of the participants were satisfied with the improvement in bloating by day 3 itself. When the data were collected on day 4 (cumulative results of the first 3 days), these participants reported significant relief in other cardinal symptoms, mainly early satiety, and postprandial fullness compared to baseline. By day 8, ASF treatment was found to offer significant relief to epigastric pain and heartburn, and by day 14, more than 75% of the participants in general reported more than 60% relief in their overall symptoms. The observed reduction in symptoms indicates improved digestive health, with a more balanced and efficient digestive system. However, the intensity of the various symptoms was different for different people at the baseline and at the end of the study. Placebo on the other hand also showed significance in some of the symptoms. The significant placebo effect in self-reported questionnaires is very common in clinical studies on dyspepsia mainly due to various psychological issues such as positive expectancy and interdependency of the symptoms.^[44]^

The relative changes observed in stool types, as evident from BSFS data, were also in agreement with the self-reported improvement in digestion. It was observed that the compliance rate in ASF group was 100% versus 92% in placebo. It was also observed that 8 participants (~30%) from ASF group consumed an additional dose (in addition to the regular dose under fasting), when they felt bloating later in the day, and reported significant relief in 1 hour. This indicated the usefulness of ASF intake during bloating and postprandial fullness symptoms, or generally for postprandial distress.

It is well documented that a healthy gut microbiome supports a well-functioning digestive system and promotes overall GI health, thereby reducing the incidence and severity of GI disorders and symptoms. Indian curry spices have already reported to modulate gut microbiota in 24 to 48 hours.^[27]^ Asafoetida is a popular Indian kitchen spice widely using in vegetable curries. So, we monitored the effect of ASF on gut microbiome and stool type, since the stool type has also been shown to be a sensitive marker for digestion and gut health. The significant differences observed in the population of *Bifidobacterium, Ruminococus, Prevotella*, and *Bacteroides* compared to the baseline (pretreatment condition) show the potential positive functional effects of ASF on GI health (Fig. 4). For instance, *Bifidobacterium* is known to relieve constipationwhich has been reflected in our BSFS results with an increase in type 3 and 4 stools from the baseline type 1 and 2.^[45]^ *Prevotella* restoration was reported to alleviate eating-related PDS symptoms.^[46]^ Similarly, the role of *Bacteroides* in producing short-chain fatty acids and facilitating dietary metabolism through the breakdown of polysaccharides and oligosaccharides, thereby providing essential nutrients and vitamins to the host and other intestinal microbes, is well documented.^[47]^ Overabundance of several potentially harmful bacterial taxa, including *Clostridia, Ruminococcus*, and *Escherichia*, was observed at the baseline of ASF group. Although many species within the *Clostridia* and *Ruminococcus* genera are known for their beneficial production of short-chain fatty acids, certain species pose health risks. For instance, *Clostridium difficile* is known for the potential to produce toxins that cause severe colitis during overgrowth.^[48,49]^ Similarly, an overabundance of *Ruminococcus* species has been linked to conditions such as obesity, inflammatory bowel disease, and irritable bowel syndrome, largely due to excessive fermentation of dietary fibers, leading to gas production and other GI disturbances.^[50,51]^ But certain *Ruminococcus* species were shown to enhance BDNF (biomarker of cognitive function) levels through serum short chain fatty acids.^[52,53]^ The ASF treatment successfully reduced the levels of these harmful taxes to baseline demonstrating its beneficial impact on gut health.

The F/B ratio is a widely recognized biomarker for gut microbiome health, with elevated levels often correlated with dysbiosis and metabolic disturbances.^[54,55]^ The marked reduction in the F/B ratio in ASF group indicates a beneficial modulation of the gut microbiota in 14 days. A lower F/B ratio is typically linked to enhanced microbial diversity and improved intestinal health, suggesting that ASF may promote the proliferation of *Bacteroides* or the suppression of *Firmicutes*. The bioactive compounds in ASF and the fenugreek soluble fiber used in the formulation are likely to exert prebiotic effects, contributing to a more balanced gut ecosystem, diminished inflammatory responses, and improved intestinal barrier integrity. *Bacteroides* strains, known for their anti-inflammatory properties, can attenuate pro-inflammatory mediators and reinforce epithelial barrier function.^[56,57]^ These findings underscore ASF potential to maintain gut health through targeted microbiome modulation.

The intricate interplay between gut microbiome and cognitive functions (MGBA) has been recently demonstrated in human beings.^[12,58]^ Though the pathogenesis of FD has not been fully clarified, it was found to be linked to central alterations affecting multiple neural networks, including those regulating visceral sensation, pain, emotion, and cognition.^[59,60]^ Studies have also highlighted the role of cognitive behavioral therapy for the management of FD.^[59]^ Reaction time and processing speed are the measurements of the cognitive function, and they serve as the indicators of attention and focus.^[41]^ Treatment and treatment × time analysis results of CRT in the present study showed a significant reduction in reaction time, indicating the improvement in attention by ASF. The observed improvement in cognitive performances may be attributed to the potential role of ASF in the modulation of the GBA. Prior research has also established that, the gut microbiome can significantly impact cognitive functions like attention, memory, and mood through the production of neuroactive metabolites and regulation of neuroinflammation.^[61,62]^

Yet another commonly reported issue associated with FD is the lack of quality sleep.^[63]^ Poor quality of sleep has been found to be closely associated with the severity of dyspepsia symptoms, which in turn affects quality of life and work productivity.^[14]^ A dysregulated GBA can lead to sleep disturbances.^[11]^ Quality of sleep was assessed by BIS, a validated questionnaire most widely used against Pittsburgh Sleep Quality Index.^[64]^ ASF group showed significant improvement in sleep quality compared to placebo (ΔBIS = 10 ± 2.61; *P* < .001). This result was also supported by treatment × time analysis, confirming the positive change in sleep quality over the time period. Previous studies have also indicated that the gut microbiota modulation can influence sleep quality by affecting neuroinflammatory and neuroendocrine signaling pathways.^[65,66]^

The fast action of ASF to alleviate dyspepsia symptoms and to improve quality of life within short duration of supplementation (14 days) may be multifactorial, involving the modulation of gut microbiome to establish a healthy GBA, and further to modulate neurotransmitters and neurotropic factors.^[28,29,32,33]^ In a preclinical study on cisplatin-induced dyspepsia model of rats, asafoetida significantly improved gut integrity, restored mucosal structure, and balanced key gut-brain hormones and neurotransmitters, including ghrelin, leptin, and serotonin.^[67]^ These results provide mechanistic insight that complements for the present study and further supports the gut-brain modulatory potential of Asafin. Moreover, asafoetida has already been reported to relax smooth muscles of the GI tract, thus helping to restore impaired gastric motor function.^[60,68]^ Smooth muscle contractility is a contributing factor to the pathophysiology of functional gastrointestinal disorders, including FD.

Yet another important observation is the lack of any side effects or adverse events, despite the gummy and unfavorable taste characteristics of asafoetida. The reason may be attributed to the formulation technology which allowed the microencapsulation of the asafoetida oleo-gum resin within the fenugreek galactomannan-based hydrogel scaffold, a prebiotic soluble dietary fiber (FenuMat technology). The technology allowed the controlled release and targeted delivery of asafoetida oleo-gum resin in the lower abdominal tract without the taste or burping issues. This is clear from the reduction in the gas-producing microbes like *Clostridium* and *Escherichia* upon ASF treatment, while supporting beneficial microbes such as *Lactobacillus* and *Bifidobacterium*. Carminative effects and improvement in gastric motility are other factors which can alleviate excessive gas production and abdominal distension.

## 5. Limitations and future directions of the study

The analysis on short-chain-fatty acids and neurotransmitters involved in gastric motility and brain functions would have added more insight to the present study. Moreover, BSFS data on a daily basis would also have been beneficial to gain more information on digestion and constipation. Future studies on the effect of ASF on *H pylori* infection and human studies in comparison with standard drugs are warranted to gain more information on its therapeutic effect. Further, the relatively short intervention period (14 days) and the use of a single fixed-dose regimen limited the ability to assess the long-term efficacy and dose–response relationship of ASF. Moreover, future studies in comparison with conventional dietary approaches such as fiber-rich foods (e.g., whole grains), also recommended to gain more information on its specific effects on digestive health.

## 6. Conclusion

In summary, the present randomized, double-blind, placebo-controlled study demonstrated the fast action of ASF in the amelioration of dyspepsia symptoms, most importantly the postprandial distress symptoms including bloating, postprandial fullness, early satiety, heart burn, and belching, with improved digestion. While the participants supplemented with ASF generally revealed satisfaction upon single dose treatment itself, the 3 days of supplementation reported significant relief from bloating-related issues (fullness and early satiety), mainly within 1 hour of ASF administration. On day 7, a greater number of participants reported relief to their heart burn, and belching issues, and by day 14, more than 90% of the participants were ready to continue the supplementation for 3 months. Neurocognitive tests on day 14 also revealed a significant improvement in focus, attention, and sleep quality. The improvement in dyspepsia symptoms and cognitive functions further correlated with the favorable modulation in gut microflora as evident from the F/B ratio, increased alpha diversity (*P* < .05), and increase in beneficial taxa and reduced harmful ones. Improvement in digestion and reduced constipation was also evident from Bristol stool type analysis. ASF was also well-tolerated with no significant adverse effects. Collectively, our results indicate the positive modulation of MGBA, and reduced dyspepsia symptoms severity scores, improved focus and sleep, and digestion upon ASF supplementation.

## Acknowledgments

The authors acknowledge BGS Global Institute of Medical Sciences in Bangalore, India for their support in the study.

## Author contributions

**Conceptualization:** Baby Chakrapani PS, Krishnakumar Illathu Madhavamenon.

**Data curation:** Veena RM, Johannah Natinga Mulakal.

**Formal analysis:** Syam Das S, Veena RM, Sherin Joy Parappilly, PA Aneesa, Johannah Natinga Mulakal.

**Funding acquisition:** Krishnakumar Illathu Madhavamenon.

**Investigation:** Veena RM, Sherin Joy Parappilly, PA Aneesa, Johannah Natinga Mulakal, Baby Chakrapani PS, Krishnakumar Illathu Madhavamenon.

**Methodology:** Syam Das S, Veena RM.

**Project administration:** Baby Chakrapani PS.

**Resources:** Syam Das S, PA Aneesa, Johannah Natinga Mulakal, Baby Chakrapani PS, Krishnakumar Illathu Madhavamenon.

**Software:** Sherin Joy Parappilly, Johannah Natinga Mulakal.

**Supervision:** Veena RM, Baby Chakrapani PS, Krishnakumar Illathu Madhavamenon.

**Validation:** Veena RM, Sherin Joy Parappilly, PA Aneesa.

**Visualization:** Johannah Natinga Mulakal.

**Writing – original draft:** Syam Das S.

**Writing – review & editing:** Baby Chakrapani PS, Krishnakumar Illathu Madhavamenon.

## Supplementary Material
