# Supplementary material for: Ferula asafoetida oleo-gum resin alleviates dyspepsia symptoms through modulation of microbiome-gut-brain axis: A randomized, double-blind, placebo-controlled study
Source: Medicine (Baltimore). 2025 Oct 3;104(40):e44590. doi: 10.1097/MD.0000000000044590 (PMC12499811; doi:10.1097/MD.0000000000044590)
Supplement: Supplementary file 1 [file medi-104-e44590-s001.docx]

**Table S1**: Intergroup comparison of LPDS sub-scores upon treatment with ASF and placebo

| **Time** | **Early satiety** | | **Postprandial fullness** | | **Bloating** | | **Belching** | | **Heart burn** | |
| --- | --- | --- | --- | --- | --- | --- | --- | --- | --- | --- |
|  | ∆Mean | *P* value | ∆Mean | *P* value | ∆Mean | *P* value | ∆Mean | *P* value | ∆Mean | *P* value |
| Day 4 | 1.05±0.01 | .001 | 1.38±0.19 | <.001 | 1.07±0.22 | <.001 | 0.65±0.10 | .007 | 0.67±0.30 | .011 |
| Day 8 | 0.84±0.21 | .005 | 1.20±0.29 | <.001 | 1.46±0.30 | <.001 | 0.90±0.01 | .001 | 0.66±0.22 | .030 |
| Day 14 | 2.05±0.33 | <.001 | 1.04±0.40 | <.001 | 1.26±0.45 | <.001 | 0.69±0.33 | .015 | 1.05±0.46 | <.001 |

Data presented in the table represents the mean change in FD symptoms among population reported with high LPDS Score (≥ 3) at baseline. Values are expressed as difference in mean ± SD and respective p-value. P-value less than 0.05 is considered as statistically significant.

LPDS = Leuven postprandial distress scale, ASF = Asafin

**Table S2**. Haematological and biochemical parameters

| **Parameter** | **Groups** | **Day 1** | **Day 14** | ***P* value** | **Normal range** |
| --- | --- | --- | --- | --- | --- |
| **Hemoglobin (g/dL)** | Placebo | 14.09 ± 0.34 | 13.75 ± 0.35 | *P* = .185 | 12 – 17.5 |
|  | ASF | 14.29 ± 0.31 | 13.81 ± 0.28 |  |  |
| **Hematocrit (%)** | Placebo | 43.52 ± 0.88 | 42.79 ± 0.92 | *P* = .356 | 36 – 54 |
|  | ASF | 44.11 ± 0.88 | 43.39 ± 0.78 |  |  |
| **RBC (10 ^6^/µL)** | Placebo | 4.63 ± 0.09 | 4.56 ± 0.09 | *P* = .168 | 4 – 6.5 |
|  | ASF | 4.62 ± 0.10 | 4.51 ± 0.09 |  |  |
| **WBC (10 ^3^/µL)** | Placebo | 6.55 ± 0.33 | 5.55 ±0.48 | *P* > .05 | 4 – 10 |
|  | ASF | 6.17 ± 0.26 | 4.69 ± 0.35 |  |  |
| **Platelet count (10 ^6^/µL)** | Placebo | 269 ± 21.89 | 238.7 ± 19.00 | *P* > .05 | 150 – 450 |
|  | ASF | 255.1 ±10.00 | 223.5 ± 11.00 |  |  |
| **SGOT (IU/L)** | Placebo | 25.46 ± 0.93 | 26.28 ± 0.93 | *P* = .267 | 8 – 45 |
|  | ASF | 27.18 ± 0.93 | 25.65 ± 0.90 |  |  |
| **SGPT (IU/L)** | Placebo | 33.27 ± 1.22 | 35.14 ± 1.21 | *P* > .05 | 7 – 56 |
|  | ASF | 38.71 ± 0.93 | 33.65 ± 1.17 |  |  |
| **Creatinine (mg/dL)** | Placebo | 0.77 ± 0.02 | 0.82 ± 0.02 | *P* = .156 | 0.7 – 1.3 |
|  | ASF | 0.81 ± 0.02 | 0.76 ± 0.02 |  |  |

Values are presented as mean ± SD. A ‘*P*’ value less than 0.05 (*P* < 0.05) was considered statistically significant.

RBC = Red blood cell; WBC = White blood cell; SGOT = Serum Glutamic-Oxaloacetic Transaminase; SGPT = Serum Glutamic Pyruvic Transaminase; ASF = Asafin
