# Supplementary material for: Ferula asafoetida oleo-gum resin alleviates dyspepsia symptoms through modulation of microbiome-gut-brain axis: A randomized, double-blind, placebo-controlled study
Source: Medicine (Baltimore). 2025 Oct 3;104(40):e44590. doi: 10.1097/MD.0000000000044590 (PMC12499811; doi:10.1097/MD.0000000000044590)
Supplement: Supplementary file 2 [file medi-104-e44590-s002.docx]

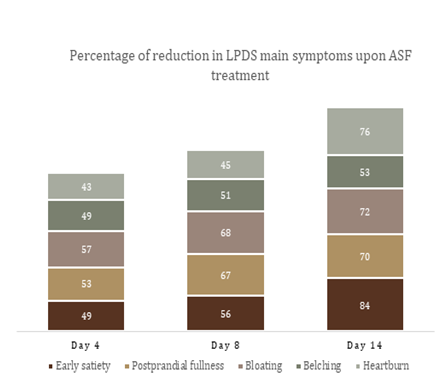


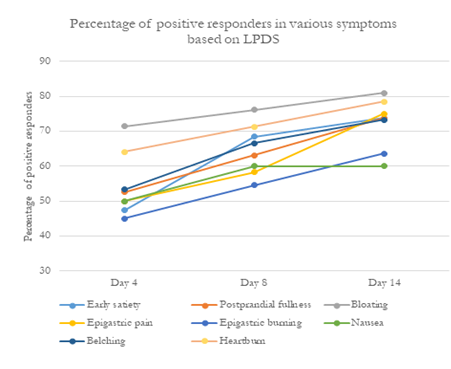


1. b)

**Figure 1.** a) Percentage of positive responders assessed by LPDS in ASF. b) Percentage of reduction in LPDS sub scores in ASF.

LPDS = Leuven Postprandial Distress Scale
